# Supplementary material for: The Interconnection Between Systemic Lupus Erythematosus and Diet: Unmet Needs, Available Evidence, and Guidance—A Patient-Driven, Multistep-Approach Study
Source: Nutrients. 2024 Nov 29;16(23):4132. doi: 10.3390/nu16234132 (PMC11643781; doi:10.3390/nu16234132)
Supplement: Supplementary file 1 [file nutrients-16-04132-s001.zip › nutrients-3307238-supplementary.pdf]

Table S1. Included studies examining the anti-inflammatory properties of foods

Study	Authors (Year)	# studies included	Pre- clinical studies	Clinical studies	Inclusion criteria	Main results
Can supplementation with vitamin D reduce the risk or modify the course of autoimmune diseases? A systematic review of the literature [42]	Antico A, et al. (2012)	219	0	219	The studies included were those that: scheduled the dosage of levels of 25(OH)D3, used for the determination of the hormone quantitative immunometric methods and chromatographic methods	Experimental studies in humans performed to date indicate the beneficial effects of vit D supplementation in reducing the severity of disease activity; it could be that the dose of hormone supplied is insufficient to control the autoimmune aggression or to prevent the onset of the disease.<br>The data present in literature deriving from the studies linking vitamin D level and the risk of developing autoimmune diseases are insufficient to establish a direct link between deficiency of the hormone and incidence of the pathology.
Dietary intervention and health in patients with systemic lupus erythematosus: A systematic review of the evidence [6]	de Medeiros M, et al. (2019)	11	0	11	Original articles were included with dietary intervention, either for food or supplements, in adults with SLE (18 to 59 years), in both sexes, published between 2006 and 2016, in English, Spanish or Portuguese.<br>The studies should report on the intervention performed and the effects on the health status of SLE<br>patients.	The studies evidenced that omega-3 supplementation reduced inflammation, disease activity, endothelial dysfunction and oxidative stress; vitamin D supplementation increased serum levels, reduced inflammatory and hemostatic markers; turmeric supplementation reduced proteinuria, hematuria and systolic blood pressure; and low glycaemic index diet caused weight loss and reduced fatigue.
Effect of omega-3 fatty acids on systemic lu-pus erythematosus disease activity: A systematic review and meta- analysis [15]	Duarte-García A, et al. (2020)	8	0	8	Adult patients (≥18 years) with SLE according to one of the accepted classification criteria.<br>All RCTs fulfilling the following criteria were included:<br>Trials compared omega-3 fatty acids supplementation to placebo or standard of care.<br>SLE disease activity (before and after intervention or a difference in pre- and post- intervention) was reported.<br>Duration of follow-up was at least 12 weeks.	Omega-3 fatty acids reduced disease activity by 0.9 SLEDAI points compared to placebo.<br>This meta-analysis suggests that omega-3 fatty acids could provide therapeutic benefit in addition to immunosuppres-sive regimens used for SLE.
Vitamin D supplementation and disease activity in patients with immune- mediated rheumatic diseases [10]	Franco A, et al. (2017)	9<br>(3 on SLE)	0	9	Types of studies: only randomized controlled trials, double-blinded or not, that studied the effect of vitamin D supplementation or its analogs were included.<br>Types of participants: trials conducted on participants with any of the following diseases were eligible: Behçet Syndrome, Dermatomyositis, Juvenile Arthritis, Mixed Connective Tissue Disease, Polymyalgia Rheumatica, Rheumatic	Vitamin D supplementation reduced anti-dsDNA positivity on systemic lupus erythematosus. Vitamin D supplementation may be beneficial to patients with high anti-dsDNA positivity, possibly reducing clinical flares.

					Fever, Rheumatoid Arthritis, Sjogren's Syndrome, Ankylosing Spondylitis, Systemic Lupus Erythematosus, Systemic Sclerosis, and Vasculitis.<br>Types of intervention: selected manuscripts were required to include studies investigating the supplementation of vitamin D or its analogs for at least 3 months. This supplementation was compared with a matching placebo or no drugs.<br>Types of outcome measures: this review in-cluded trials that studied appropriate clinical and/or laboratory outcomes related to disease activity for each disease before and after vitamin D supplementation.	
Association between circulating 25- hydroxyvitamin D and systemic lupus erythematosus: A systematic review and meta-analysis [8]	Guan S, et al. (2019)	19	0	19	Cross-sectional, case-control or cohort studies; associations between 25(OH)D and SLE were reported; studies were published in English.	No matter the changes of age, disease duration, and the therapy of corticosteroid or immuno-suppressive or neither, circulating 25 (OH)D levels were sig-nificantly reduced in SLE patients. The deficiency, insufficiency and sufficiency of vitamin D could significantly ele-vate, slightly decrease (not significantly), significantly decrease SLE risk, respectively.
Effects of diet on the outcomes of rheumatic and musculoskeletal diseases (RMDs): Systematic review and meta-analyses informing the 2021 EULAR recommendations for lifestyle improvements in people with RMDs [37]	Gwinnutt J, et al. (2022)	174<br>(24 SRs, 150 OAs)	0	174	Systematic reviews or meta-analyses of randomised controlled trials (RCTs) or observational studies.<br>Including people with an RMD (OA, RA, SLE, axSpA, PsA, SSc, gout).<br>Studying the relationship between diet and outcomes.<br>Longitudinal study design (randomised trials, non-randomised trials, single-arm intervention studies, longitudinal observational studies).<br>Including adults with an RMD (OA, RA, SLE, axSpA, PsA, SSc, gout).<br>Studying the relationship between dietary expo-sures and outcomes.	High consumption of vitamin B6, fibre and vitamin C was asso-ciated with lower risk of developing active disease. Two out of three studies included in the systematic review reported reductions in disease activity following omega-3 intervention. The evidence for fish oil/omega- 3 for SLE was rated as moderate but showed no effect on outcomes
Effects of Vitamin D on Systemic Lupus Erythematosus Disease Activity and Autoimmunity: A Systematic Review and Meta-Analysis [5]	Irfan S, et al. (2022)	6	0	6	Population: diagnosed SLE patients<br>Exposure: vitamin D supplementation<br>Control: baseline characteristics<br>Outcome: effectiveness of vitamin D on disease activity and autoimmunity<br>Studies: RCTs.	Vitamin D in SLE patients showed a significant decrease in SLEDAI scores and a significant increase in C3 levels. The effect of vitamin D on fatigue was inconclusive. No significant dif-ference in anti-dsDNA and C4 levels was noted.
Immunomodulatory Effects of Diet and Nutrients in Systemic Lupus Erythematosus (SLE): A Systematic Review [25]	Islam M, et al. (2020)	184	/	/	Only English-language articles were searched. There was no year restriction, and the final systematic search was conducted on 22 December 2019. Review articles, non-English articles, errata, letters, comments, editorials, and duplicate articles among	Based on the currently published literature, it was observed that a low-calorie and low-protein diet with high contents of fiber, polyunsatu-rated fatty acids, vitamins, minerals and polyphenols contain sufficient potential macronutrients and micronutrients to regulate the activity of the

					different databases were excluded.	overall disease by modulating the inflammation and immune functions of SLE.
Vitamin D status in patients with systemic lupus erythematosus (SLE): A systematic review and meta- analysis [7]	Islam M, et al. (2019)	34	0	34	Case-control studies assessing the serum levels of vitamin D in patients with SLE of adult age (≥18 years), of any sex or race were considered eligible patients. Healthy subjects without the history of any autoimmune disorders including SLE of adult age (≥18 years), any sex or race were considered eligible control participants.	Serum levels of vitamin D was detected significantly lower in the SLE patients compared to healthy controls.
Diet and Systemic Lupus Erythematosus (SLE): From Supplementation to Intervention [ref?]	Jiao H, et al. (2022)	14	0	14	Intervention studies and randomised controlled trials, blind or not on adult patients diagnosed with SLE, applying dietary interventions through dietary supplements or specific dietary patterns, and including a control group.<br>Eligible studies included a control (no-intervention) or control or comparison group (other type of intervention) and compared the effects of the intervention versus the control/comparison group. Studies reporting the effects of the intervention on disease activity, clinical parameters, and health status of patients with SLE.	Vitamin D or E supplementation was associated with respective improvement of inflammatory markers or antibody production, but not disease activity scores in most studies.<br>Despite their expected synergistic actions, the addition of curcumin on vitamin D supplementation had no additional effects on disease activity or inflammatory markers. Trials of omega-3 fatty acid supplementation presented significant reductions in ESR, CRP, disease activity, inflammatory markers, and oxidative stress, and improved lipid levels and endothelial function.<br>A low glycemic index (GI) diet showed evidence of reduced weight and improved fatigue in patients.
Curcumin, autoimmune and inflammatory diseases: going beyond conventional therapy–a systematic review [19]	Marton L, et al. (2022)	36 (2 on SLE)	0	36	Randomized Clinical Trials that discussed the use of curcumin and its effects on autoimmune and inflammatory diseases.<br><br>Only articles written and published in English from the last ten years that showed correspondence with the keywords were selected.	A significant decrease in proteinuria, systolic pressure, and hematuria were met when comparing pre and 1, 2, and 3 months supplementation in the trial group. No adverse effect related to turmeric was observed during the trial. The use of short-term supplementation with turmeric is associated with benefits in subjects with relapsing or refractory lupus nephritis and could be used as safe adjuvant therapy.<br><br>There were no significant differences in SLEDAI, IL- 6, and TGF-β serum among groups after the treatment.<br><br>The curcumin doses that brought benefits to the patient with lupus ranged from 20 to 500 mg (orally administrated).
The impact of curcumin supplementation on systemic lupus erythematosus and lupus nephritis: A	Ramessar N, et al. (2023)	13	10	3	The inclusion criteria for this review were full research articles, articles written in English, trials conducted in human and mice subjects, and trials	In human trials, curcumin decreased 24-h and spot proteinuria, but the trials were small, ranging from 14 to 39 patients, with varied curcumin doses

systematic review [18]					that assessed the impact of curcumin or turmeric supplementation on SLE.	and different study dura-tions ranging from 4 to 12 weeks.<br><br>There was no change in C3, dsDNA, or the Systemic Lupus Erythematosus Disease Activity (SLEDAI) scores even in the longer trials.<br><br>In the mouse models NF-κβ activation was suppressed along with inducible nitric oxide synthase (NOS) species expression when 1 mg/kg/day of curcumin was administered for 14 weeks, leading to sig-nificant decreases in dsDNA, proteinuria, renal inflammation, and IgG subclasses. A reduction in pro- inflammatory Th1 and Th17 percentages, IL-6 and anti- nuclear antibody (ANA) levels were reported.
The effect of Omega-3 fatty acid supple-mentation in systemic lupus erythematosus patients: A systematic review [14]	Ramessar N, et al. (2022)	13	0	13	Full research articles, English articles, human trials, and trials that as-sessed the impact of fish oil or omega-3 fatty acids on SLE published from 1 January 1980 to 1 April 2021.	Current data show that there is a potential benefit on disease activity as demonstrated by SLAM-R, Systemic Lupus Er-ythematosus Disease Activity Index (SLEDAI), and British Isles Lupus Assessment Group (BILAG) scores and plasma membrane arachidonic acid composition and urinary 8- isoprostane levels, with mini-mal adverse events.
The Key Role of Glutathione Compared to Curcumin in the Management of Systemic Lupus Erythematosus: A Systematic Review [40]	Ravi N, et al. (2022)	15	0	15	Papers included in this study date from 2002 to 2022. The English lan-guage is a filter for selecting patients with SLE using either glutathione or curcumin. Only human studies are part of this study.	Curcumin and glutathione can act as potent drugs for treating lupus. Curcumin can be a more promising alternative since it operates on various pathways and is a more easily accessible source.
Correlation between serum 25(OH)D values and lupus disease activity: An original article and a systematic review with meta- analysis focusing on serum Vit D confounders [41]	Sahebari M, et al. (2014)	38 (11 included in the meta-analysis)	0	38	Eligible for inclusion were observational studies of all designs, published in any language that pre-sented an association between serum values of VitD and SLE disease activity.<br><br>The total number of pa-tients in the selected ar-ticles should be more than 20 patients.	According to the results of this meta-analysis Vit D correlates inversely with disease activity in lupus.
The Clinical Significance of Vitamin D in Systemic Lupus Erythematosus: A Systematic Review [9]	Sakthiswary R, Raymond A. (2013)	22	0	22	All adult human cohort and case-control studies written in English, which investigated the role and effects of vitamin D in SLE published between the years 2000 and 2012 were included.	The association between vitamin D and SLE disease activity revealed a statistically significant inverse relationship.<br><br>There is convincing evidence to support the association between vitamin D levels and SLE disease activity.
Bone mineral density and vitamin D status in systemic lupus ery-thematosus (SLE):	Salman- Monte T, et al. (2017)	Ns	Ns	Ns	Included all intervention studies and observational studies in which vitamin D plasma levels, BMD and bone loss were measured and applied to	SLE patients are at risk for de-veloping reduced vitamin D plasma levels and low BMD; it is therefore essential to study, monitor, prevent

A systematic review [11]					patients with SLE.	and treat bone metabolism disorders in SLE patients.
Effect of vitamin D supplementation on patients with systemic lupus erythematosus: a systematic review [43]	Sousa J, et al. (2017)	4	0	4	(P) Patients with systemic lupus erythematosus<br><br>(I) Vitamin D supplementation<br><br>(C) Placebo<br><br>(O) Clinical improvement of SLE patients.<br><br>Having a controlled clinical trial design and being available as a full-text article.	The results of three studies showed a positive effect of supplementation on disease activity reduction and significant improvement in levels of inflammatory markers, fatigue, and endothelial function.<br><br>The data from this review provide evidence on the benefits of vitamin D supplementation in patients with lupus and vitamin D insufficiency/deficiency.
Serum Homocysteine, Folate, and Vitamin B12 Levels in Patients with Systemic Lupus Erythematosus: A Meta- Analysis and Meta- Regression [27]	Tsai T, et al. (2021)	50	0	50	Observational studies comparing the serum lev-els of homocysteine, folate, and vitamin B12 between patients with SLE and those without (control group).	Serum homocysteine levels were higher and vitamin B12 levels were lower among individuals with SLE than those without SLE.<br><br>The meta-regression analysis reported inverse correlations between the SMD of homocysteine and C3/C4 levels. Current evidence for supple-menting these patients with vitamin B12 and/or folate is still insufficient.
Curcumin and Curcuma longa Extract in the Treatment of 10 Types of Autoimmune Dis-eases: A Systematic Review and Meta-Analysis of 31 Randomized Controlled Trials [39]	Zeng L, et al. (2022)	34<br>(2 on SLE)	0	34	Participants: the patient was diagnosed with an autoimmune disease by accepted diagnostic criteria. Intervention: the therapy of the experimental group was curcumin or Curcuma longa Extract preparation, regardless of dosage form, intervention dose, administration route, etc. The therapy of the control group was placebo, conventional therapy, or other curcumin-free therapy. Outcomes: efficacy indicators, inflammatory indicators and safety indicators. Design: the study design was RCTs.	Curcumin may enhance regulatory responses involving T reg cells by inhibiting antibody-antigen interactions, reducing autoantigen-autoantibody deposition in tissues and various microvascular beds, and inhibiting antibody production. However, more RCTs are needed to further verify the therapeutic effect and safety of curcumin on SLE.
Legend: SLE Systemic Lupus Erithematosus; SR systematic review; OA original article; Ns not specifyed.						

Table S2. Included studies examining the possibilities for the dietary regimen of reducing/avoiding flares

Study	Authors (Year)	# studies included	Pre- clinical studies	Clinical studies	Inclusion criteria	Main results
Immunomodulatory Effects of Diet and Nutrients in Systemic Lupus Erythematosus (SLE): A Systematic Review [25]	Islam M, et al. (2020)	184	/	/	Only English-language articles were searched. There was no year restriction, and the final systematic search was conducted on 22 December 2019. Review articles, non-English articles, errata, letters, comments, editorials, and duplicate articles among different databases were excluded.	Based on the currently published literature, it was observed that a low-calorie and low-protein diet with high contents of fiber, polyunsatu-rated fatty acids, vitamins, minerals and polyphenols contain sufficient potential macronutrients and micronutrients to regulate the activity of the overall disease by modulating the inflammation and immune functions of SLE.
Healthy lifestyle habits for patients with systemic lupus erythematosus: A systemic review [33]	Rodríguez Huerta M, et al. (2016)	21	0	21	Experimental, quasi- ex-perimental, and observa-tional studies published in English or Spanish, which evaluated the effects of life-style habits on SLE adults (≤ 18 years) were included.<br>Outcome measures consid-ered were clinical (e.g., dis-ease activity and damage, physical function) and/or patient-reported outcomes (e.g., fatigue and HRQoL)	Tobacco smoking increases the risk of skin damage and disease ac-tivity in patients with SLE. A diet rich in polyunsaturated fatty acids, avoiding a sedentary lifestyle and supervised exercise should be recommended for patients with stable SLE.
Smoking, alcohol con-sumption and disease- specific outcomes in rheumatic and musculo-skeletal diseases (RMDs): systematic reviews in-forming the 2021 EULAR recommendations for life-style improvements in people with RMDs [32]	Wieczorek M ,et al. (2022)	90	0	90	Systematic reviews were eligible if (1) the study pop-ulation involved people with an RMD (OA, RA, SLE, axSpA, PsA, SSc, gout), (2) the aim was to assess the relationship between lifestyle exposures (diet, exercise, weight, smoking, alcohol, work) and (3) data on out-comes of interest was re-ported.<br>Individual studies were eli-gible if (1) the exposure studies was smoking or al-cohol consumption, (2) the study population involved people with an RMD (OA, RA, SLE, PsA, SSc, gout (and axSpA for the alcohol re-view)), (3) the study design was longitudinal (random-ised controlled trials, non-randomised trials, single-arm intervention studies, lon-gitudinal observational studies) and (4) the aim was to investigate the relationship between smoking or alcohol and outcomes of interest.	Among SLE patients, smokers also tended to have worse outcomes, for example, worse scores on SF-36 mental and physical domains, more rashes, worse disease activity and more CV morbidity.

Legend: SLE Systemic Lupus Erithematosus; SR systematic review; OA original article; Ns not specifyed.

Table S3. Included studies examining the supplements

Study	Authors (Year)	# studies included	Pre- clinical studies	Clinical studies	Inclusion criteria	Main results
Can supplementation with vitamin D reduce the risk or modify the course of autoimmune diseases? A systematic review of the literature [42]	Antico A, et al. (2012)	219	0	219	The studies included were those that: scheduled the dosage of levels of 25(OH)D3, used for the determination of the hormone quantitative immunometric methods and chromatographic methods.	Experimental studies in humans performed to date indicate the beneficial effects of vit D supplementation in reducing the severity of disease activity; it could be that the dose of hormone supplied is insufficient to control the autoimmune aggression or to prevent the onset of the disease.<br>The data present in literature deriving from the studies linking vita-min D level and the risk of developing autoimmune diseases are insufficient to establish a direct link between deficiency of the hormone and incidence of the pathology.
Dietary intervention and health in patients with systemic lupus erythematosus: A systematic review of the evidence [6]	de Medeiros M, et al. (2019)	11	0	11	Original articles were in-cluded with dietary in-tervention, either for food or supplements, in adults with SLE (18 to 59 years), in both sexes, published between 2006 and 2016, in English, Spanish or Portuguese. The studies should report on the in-tervention performed and the effects on the health status of SLE patients.	The studies evidenced that omega-3 supplementation reduced inflam-mation, disease activity, endothe-lial dysfunction and oxidative stress; vitamin D supple-mentation increased serum levels, reduced inflammatory and hemostatic markers; turmeric supplementation reduced proteinuria, hematuria and systolic blood pressure; and low glycaemic index diet caused weight loss and reduced fatigue.
Effect of omega-3 fatty acids on systemic lupus erythematosus disease activity: A systematic review and meta-analysis [15]	Duarte-García A, et al. (2020)	8	0	8	Adult patients (≥18 years) with SLE according to one of the accepted classifi-cation criteria.<br>All RCTs fulfilling the following criteria were included:<br>- Trials compared omega-3 fatty acids supplementa-tion to placebo or standard of care.<br>- SLE disease activity (before and after in-tervention or a difference in pre- and post- intervention) was reported.<br>- Duration of fol-low-up was at least 12 weeks.	Omega-3 fatty acids reduced disease activity by 0.9 SLEDAI points compared to placebo.<br>This meta-analysis suggests that omega-3 fatty acids could provide therapeutic benefit in addition to immunosuppressive regimens used for SLE.
Vitamin D supplementation and disease activity in patients with immune- mediated rheumatic diseases [10]	Franco A, et al. (2017)	9 (3 on SLE)	0	9	Types of studies: only randomized controlled trials, double-blinded or not, that studied the effect of vitamin D supple-mentation or its analogs were included.<br>Types of participants: trials conducted on par-ticipants with any of the following diseases were eligible: Behçet Syn-drome, Dermatomyositis, Juvenile Arthritis, Mixed Connective Tissue Dis-ease, Polymyalgia Rheumatica, Rheumatic Fever, Rheumatoid Ar-thritis, Sjogren's Syn-drome, Ankylosing Spondylitis, Systemic	Vitamin D supplementation reduced anti-dsDNA positivity on systemic lupus erythematosus.<br>Vitamin D supplementation may be beneficial to patients with high anti- dsDNA positivity, possibly reducing clinical flares.

					Lupus Erythematosus, Systemic Sclerosis, and Vasculitis.<br>Types of intervention: selected manuscripts were required to include studies investigating the supplementation of vitamin D or its analogs for at least 3 months. This supplementation was compared with a matching placebo or no drugs.<br>Types of outcome measures: this review in-cluded trials that studied appropriate clinical and/or laboratory outcomes re-lated to disease activity for each disease before and after vitamin D supple-mentation.	
Association between circulating 25- hydroxyvitamin D and systemic lupus erythematosus: A systematic review and meta-analysis [8]	Guan S, et al. (2019)	19	0	19	Cross-sectional, case-control or cohort studies; associations between 25(OH)D and SLE were reported; studies were published in English.	No matter the changes of age, disease duration, and the therapy of corticosteroid or immunosup-pressive or neither, circulating 25(OH)D levels were significantly reduced in SLE patients. The de-ficiency, insufficiency and suffi-ciency of vitamin D could sig-nificantly elevate, slightly decrease (not significantly), significantly decrease SLE risk, respectively.
Immunomodulatory Effects of Diet and Nutrients in Sys-temic Lupus Ery-thematosus (SLE): A Systematic Review [25]	Islam M, et al. (2020)	184	/	/	Only English-language articles were searched. There was no year re-striction, and the final systematic search was conducted on 22 December 2019. Review articles, non-English articles, errata, letters, comments, editorials, and duplicate articles among different databases were excluded.	Based on the currently published literature, it was observed that a low-calorie and low-protein diet with high contents of fiber, pol-yunsaturated fatty acids, vitamins, minerals and polyphenols contain sufficient potential macronutrients and micronutrients to regulate the activity of the overall disease by modulating the inflammation and immune functions of SLE.
Vitamin D status in patients with systemic lupus erythematosus (SLE): A systematic review and meta- analysis [7]	Islam M, et al. (2019)	34	0	34	Case-control studies as-sessing the serum levels of vitamin D in patients with SLE of adult age (≥18 years), of any sex or race were considered eli-gible patients. Healthy subjects without the history of any autoim-mune disorders including SLE of adult age (≥18 years), any sex or race were considered eligible control participants.	Serum levels of vitamin D was detected significantly lower in the SLE patients compared to healthy controls.
Diet and Systemic Lupus Erythematosus (SLE): From Supplementation to Intervention [4]	Jiao H, et al. (2022)	14	0	14	Intervention studies and randomised controlled trials, blind or not on adult patients diagnosed with SLE, applying dietary interventions through dietary supplements or specific dietary patterns, and including a control group.<br>Eligible studies included a control (no-intervention) or control or comparison group (other type of in-tervention) and compared the effects of the inter-vention versus the con-trol/comparison group.	Vitamin D or E supplementation was associated with respective improvement of inflammatory markers or antibody production, but not disease activity scores in most studies.<br>Despite their expected synergistic actions, the addition of curcumin on vitamin D supplementation had no additional effects on disease activity or inflammatory markers.<br>Trials of omega-3 fatty acid sup-plementation

					Studies reporting the ef-fects of the intervention on disease activity, clin-ical parameters, and health status of patients with SLE.	presented significant reductions in ESR, CRP, disease activity, inflammatory markers, and oxidative stress, and improved lipid levels and endothelial func-tion.<br>A low glycaemic index (GI) diet showed evidence of reduced weight and improved fatigue in patients.
The impact of curcumin supplementation on systemic lupus erythematosus and lupus nephritis: A systematic review [18]	Ramessar N, et al. (2023)	13	10	3	The inclusion criteria for this review were full re-search articles, articles written in English, trials conducted in human and mice subjects, and trials that assessed the impact of curcumin or turmeric supplementation on SLE.	In human trials, curcumin decreased 24-h and spot proteinuria, but the trials were small, ranging from 14 to 39 patients, with varied curcumin doses and different study durations ranging from 4 to 12 weeks.<br>There was no change in C3, dsDNA, or the Systemic Lupus Erythematosus Disease Activity (SLEDAI) scores even in the longer trials.<br>In the mouse models NF-κβ activation was suppressed along with inducible nitric oxide synthase (NOS) species expression when 1 mg/kg/day of curcumin was ad-ministered for 14 weeks, leading to significant decreases in dsDNA, proteinuria, renal inflammation, and IgG subclasses. A reduction in pro- inflammatory Th1 and Th17 per-centages, IL-6 and anti-nuclear antibody (ANA) levels were re-ported.
The effect of Omega-3 fatty acid supple-mentation in systemic lupus erythematosus patients: A systematic review [14]	Ramessar N, et al. (2022)	13	0	13	Full research articles, English articles, human trials, and trials that as-sessed the impact of fish oil or omega-3 fatty acids on SLE published from 1 January 1980 to 1 April 2021.	Current data show that there is a potential benefit on disease activity as demonstrated by SLAM-R, Systemic Lupus Erythematosus Disease Activity Index (SLEDAI), and British Isles Lupus Assessment Group (BILAG) scores and plasma membrane arachidonic acid com-position and urinary 8-isoprostane levels, with minimal adverse events.
Correlation between serum 25(OH)D values and lupus disease activity: An original article and a systematic review with meta- analysis focusing on serum Vit D confounders [41]	Sahebari M, et al. (2014)	38 (11 included in meta- analysis)	0	38	Eligible for inclusion were observational studies of all designs, published in any language that presented an association between serum values of VitD and SLE disease activity.<br>The total number of patients in the selected articles should be more than 20 patients.	According to the results of this meta- analysis Vit D correlates inversely with disease activity in lupus.
Effect of vitamin D supplementation on patients with systemic lupus erythematosus: a systematic review [43]	Sousa J, et al. (2017)	4	0	4	(P) Patients with systemic lupus erythematosus<br>(I) Vitamin D supple-mentation<br>(C) Placebo<br>(O) Clinical improvement of SLE patients.<br>Having a controlled clinical trial design and being available as a full-text article.	The results of three studies showed a positive effect of supplementation on disease activity reduction and significant improvement in levels of inflammatory markers, fatigue, and endothelial function.<br>The data from this review provide evidence on the benefits of vitamin D supplementation in patients with lupus and

						vitamin D insufficiency/deficiency.
Optimal management of fatigue in patients with systemic lupus erythematosus: A systematic review [44]	Yuen H, Cunningham M. (2014)	26	0	26	Publications that were in the English language, with full text available, the majority of patients diagnosed with SLE, and adults. Only intervention and observational studies reporting a fatigue or vitality measure as one of the primary or secondary outcomes were included.	Based on the studies reported in the literature, we identified nine intervention strategies that have the potential to alleviate fatigue in patients with SLE. Of the nine strategies, aerobic exercise and belimumab seem to have the strongest evidence of treatment efficacy. N-acetylcysteine and ul-traviolet-A1 phototherapy demonstrated low-to-moderate levels of evidence. Psychosocial interventions, dietary manipulation (low calorie or glycemic index diet) aiming for weight loss, vitamin D supplementation, and acupuncture all had weak evidence. Dehydroepiandrosterone is not recommended due to a lack of evidence for its efficacy.
Legend: SLE Systemic Lupus Erithematosus; SR systematic review; OA original article; Ns not specifyed.						

Table S4. Included studies examining the potential interferences between foods and therapies

Study	Authors (Year)	# studies included	Pre- clinical studies	Clinical studies	Inclusion criteria	Main results
Warfarin and food, herbal or dietary supplement interactions: A systematic review [45]	Tan C, Lee S. (2021)	149	0	149	Articles were eligible for evaluation if they had reported information regarding interactions in human subjects.	While most food, herbs and supplements can be safely taken in moderation, healthcare professionals should be aware of the increased risk of bleeding when taking several food and herbs. These include Chinese wolfberry, chamomile tea, cannabis, cranberry, chitosan, green tea, Ginkgo biloba, ginger, spinach, St. John's Wort, sushi and smoking tobacco.
Legend: SLE Systemic Lupus Erithematosus; SR systematic review; OA original article; Ns not specifyed.						

Table S5. Included studies examining the benefits deriving from low-calories or low-glycemic diet

Study	Authors (Year)	# studies included	Pre- clinical studies	Clinical studies	Inclusion criteria	Main results
Management of cardiovascular risk in systemic lupus erythematosus: A systematic review [46]	Andrades C, et al. (2017)	19	0	19	Type of study: meta-analysis, systematic reviews, randomized controlled trials, phase II, III and IV, non-randomized and uncon-trolled clinical trials, and observational studies. Types of participants: adult patients with SLE or patients with secondary antiphospholipid syndrome (APS) to SLE. Types of interventions: studies evaluating the effectiveness of interventions for health promotion, treatment of classic cardiovascular risk factors or treatment of CVRFs directly related to SLE (antimalarials, glucocorticoids, immunosuppressants). Types of outcomes: fatal and non-fatal cardiovascular events. As secondary endpoints, mortality and changes in major CVRF factors, such as systolic/diastolic blood pressure, body mass index (BMI), waist circumference, coronary calcium, glucose levels, insulin resistance (HOMA-IR), low-density lipoprotein (LDL) cholesterol, high-density lipoprotein (HDL) cholesterol, flow-mediated dilation (FMD) and smoking cessation were included.	Low-calorie and/or low glycaemic index calories may be a useful option for secondary prevention in obese patients with systemic lupus erythematosus, and exercise would be useful in improving the endothelial function measured by flow-mediated dilation in this group of patients.
The impact of a low- calorie, low-glycemic diet on systemic lupus erythematosus: a systematic review [24]	Imoto A, et al. (2021)	3	0	3	Type of patient: systemic lupus erythematosus pa-tients, with no age and gender restriction. Type of intervention: Calorie restricted (Low Cal), low glycaemic index (Low GI) or other diet involving the joint adequacy of these aspects. Control: Placebo or different types of diet. Outcome: Questionnaires and scores to evaluate the disease activity, fatigue, quality of sleep, quality of life, and lipid profile parameters. Type of study: Randomized clinical trials.	The diet had a positive impact on the quality of life and a possible clinical relevance on lipid profile. The type of diet did not affect the fatigue, quality of sleep, and disease activity as assessed through the British Isles Lupus Assessment Group. However, when the European Community Lupus Activity Measure evaluated the low gly-cemic index diet, its effect was favourable on fatigue.
Immunomodulatory Effects of Diet and Nutrients in Systemic Lupus Erythematosus (SLE): A Systematic Review [25]	Islam M, et al. (2020)	184	/	/	Only English-language ar-ticles were searched. There was no year restriction, and the final systematic search was conducted on 22 De-cember 2019. Review arti-cles, non-English articles, errata, letters, comments, editorials, and duplicate articles among different databases were excluded.	Based on the currently pub-lished literature, it was observed that a low-calorie and low-protein diet with high contents of fiber, polyunsaturated fatty acids, vitamins, minerals and polyphenols contain sufficient potential macronutrients and micronu-trients to regulate the activity of the overall disease by modulating the inflammation and immune functions of SLE.

Optimal management of fatigue in patients with systemic lupus erythematosus: A systematic review [44]	Yuen H, Cunningham M. (2014)	26	0	26	Publications that were in the English language, with full text available, the majority of patients diagnosed with SLE, and adults. Only intervention and ob-servational studies reporting a fatigue or vitality measure as one of the primary or secondary outcomes were included.	Based on the studies reported in the literature, we identified nine intervention strategies that have the potential to alleviate fatigue in patients with SLE. Of the nine strategies, aerobic exercise and belimumab seem to have the strongest evidence of treatment efficacy. N-acetylcysteine and ultraviolet-A1 phototherapy demonstrated low-to-moderate levels of evidence. Psychosocial interventions, dietary manipula-tion (low calorie or glycemic index diet) aiming for weight loss, vitamin D supplementation, and acupuncture all had weak evi-dence. Dehydroepiandrosterone is not recommended due to a lack of evidence for its efficacy.
Legend: SLE Systemic Lupus Erithematosus; SR systematic review; OA original article; Ns not specifyed.						
